# Serum amyloid A primes microglia for ATP-dependent interleukin-1β release

**DOI:** 10.1186/s12974-018-1205-6

**Published:** 2018-05-26

**Authors:** Laura Facci, Massimo Barbierato, Morena Zusso, Stephen D. Skaper, Pietro Giusti

**Affiliations:** Department of Pharmaceutical and Pharmacological Sciences, University of Padua, Largo “E. Meneghetti” 2, 35131 Padua, Italy

**Keywords:** Microglia, Serum amyloid A, Interleukin-1β, Toll-like receptor, P2X purinoceptor 7, NLRP3 inflammasome, Neuroinflammation

## Abstract

**Background:**

Acute-phase response is a systemic reaction to environmental/inflammatory insults and involves production of acute-phase proteins, including serum amyloid A (SAA). Interleukin-1β (IL-1β), a master regulator of neuroinflammation produced by activated inflammatory cells of the myeloid lineage, in particular microglia, plays a key role in the pathogenesis of acute and chronic diseases of the peripheral nervous system and CNS. IL-1β release is promoted by ATP acting at the purinergic P2X_7_ receptor (P2X_7_R) in cells primed with toll-like receptor (TLR) ligands.

**Methods:**

Purified (> 99%) microglia cultured from neonatal rat cortex and cerebellum were first primed with the putative TLR4/TLR2 agonist SAA (recombinant human Apo-SAA) or the established TLR4 agonist lipopolysaccharide (LPS) followed by addition of ATP. Expression of genes for the NLRP3 inflammasome, IL-1β, tumor necrosis factor-α (TNF-α), and SAA1 was measured by quantitative real-time polymerase chain reaction (q-PCR). Intracellular and extracellular amounts of IL-1β were determined by ELISA.

**Results:**

Apo-SAA stimulated, in a time-dependent manner, the expression of NLRP3, IL-1β, and TNF-α in cortical microglia, and produced a concentration-dependent increase in the intracellular content of IL-1β in these cells. A 2-h ‘priming’ of the microglia with Apo-SAA followed by addition of ATP for 1 h, resulting in a robust release of IL-1β into the culture medium, with a concomitant reduction in its intracellular content. The selective P2X_7_R antagonist A740003 blocked ATP-dependent release of IL-1β. Microglia prepared from rat cerebellum displayed similar behaviors. As with LPS, Apo-SAA upregulated SAA1 and TLR2 mRNA, and downregulated that of TLR4. LPS was less efficacious than Apo-SAA, perhaps reflecting an action of the latter at TLR4 and TLR2. The TLR4 antagonist CLI-095 fully blocked the action of LPS, but only partially that of Apo-SAA. Although the TLR2 antagonist CU-CPT22 was inactive against Apo-SAA, it also failed to block the TLR2 agonist Pam_3_CSK_4_.

**Conclusions:**

Microglia are central to the inflammatory process and a major source of IL-1β when activated. P2X_7_R-triggered IL-1β maturation and export is thus likely to represent an important contributor to this cytokine pool. Given that SAA is detected in Alzheimer disease and multiple sclerosis brain, together with IL-1β-immunopositive microglia, these findings propose a link between P2X_7_R, SAA, and IL-1β in CNS pathophysiology.

**Electronic supplementary material:**

The online version of this article (10.1186/s12974-018-1205-6) contains supplementary material, which is available to authorized users.

## Background

Inflammatory conditions are marked by the production of mediators such as cytokines, chemokines, reactive oxygen species, and acute phase proteins that are key elements of the accompanying physiological and metabolic changes. C-reactive protein, complement proteins, and serum amyloid A protein (SAA) are some of the principal acute phase proteins, mainly generated in the liver and released into the systemic circulation in response to inflammation [1, 2]. SAA is the generic name of a family of proteins that share high levels of sequence homology but are encoded by different genes [3]. Humans possess four SAA genes (SAA1, SAA2, SAA3, and SAA4) mapped in a 150-kb region of chromosome 11p15.115. Mice also have four SAA genes whose protein products are highly homologous to their human counterparts [3]. Inducible expression is characteristic of all acute-phase SAAs including SAA1 and SAA2. Extra-hepatic expression of SAA has been reported as well [4].

Central nervous system (CNS) disorders are characterized by central activation of innate immunity and activation of a potent peripheral acute phase response that influences central inflammation and contributes to poor outcome [5]. Syrian hamsters injected systemically with lipopolysaccharide (LPS) had elevated levels of mRNA for Apo-SAA in all tissues examined, including brain [6]. While not detectable in normal brain, SAA protein has been found in Alzheimer disease (AD) brain, along with SAA gene expression in multiple sclerosis (MS) brain tissue [7]. Elevated SAA concentration was described in cerebrospinal fluid of AD subjects [8], as well as SAA immunoreactivity that co-localized with amyloid β-peptide deposits in AD brain [9]. Induction of a systemic acute phase response in SAA transgenic mice enhanced amyloid β-peptide deposition [10]. Further, Chung et al. [11] reported a much stronger immunostaining of SAA in brain of patients with neurologically confirmed AD and MS in comparison to unaffected regions and non-AD/MS brain. Barbierato et al. [12] recently demonstrated that cortical glia responds to pro-inflammatory agents (LPS, tumor necrosis factor alpha (TNF-α), Apo-SAA) by upregulating their expression of *Saa1*.

Interleukin-1β (IL-1β), a master regulator of neuroinflammation [13] mainly produced by activated inflammatory cells of the myeloid lineage [14], contributes importantly to cellular activation and cytokine production. IL-1β plays a key role in the pathogenesis of acute and chronic diseases of both the peripheral nervous system and CNS [15–17]. LPS, a potent stimulus for IL-1β synthesis by microglia is rather inefficient, given that most of the secreted cytokine remains in the immature (inactive) pro-form [18]. One of the molecules mainly involved in IL-1β maturation is the purinoceptor P2X_7_ (P2X_7_R), an ATP-gated ion channel that chiefly acts through the recruitment of the NLRP3 inflammasome-caspase-1 complex [14, 19]. This activation process involves first recognition by toll-like receptors (TLRs, a sub-family of pattern recognition receptors) of exogenous (e.g., bacterial- and virus-derived pathogens) or endogenous (e.g., components of cell damage) stimuli to induce transcription and translation of IL-1β (‘priming’). This is followed by a secondary signal such as ATP to trigger formation of the inflammasome complex that leads to caspase 1 activation and cleavage/release of IL-1β [20–22]. P2X_7_R-triggered IL-1β maturation and export may thus represent a major contributor to this cytokine pool [20, 23].

SAA appears to be an endogenous ligand for both TLR4 [24–27] and TLR2 [28–31], despite having little structural resemblance to the bacteria-derived ligands of either receptor. Although SAA can upregulate the NLRP3 inflammasome in peripheral immune cells [25] and provoke mediator production in a variety of non-neural cells, nothing is known about its ability to stimulate IL-1β release from CNS glia in the presence of ATP, a multi-target danger signal in the brain [32] in a P2X_7_R-dependent manner. This is especially important, given the growing body of data indicating that genetic or pharmacological manipulation of P2X_7_Rs alters responsiveness in animal models of CNS neurological disorders [33]. Recent studies suggest also that P2X_7_Rs regulate the pathophysiology of psychiatric disorders, including mood disorders [33]. The present study was undertaken to examine the ability of ATP to promote the intracellular production, and release, of IL-1β from cortical microglia stimulated with Apo-SAA, and the involvement of P2X_7_R, TLR4, and TLR2.

## Methods

Tissue culture media, antibiotics, fetal calf serum (FCS), and NP40 cell lysis buffer (10×) were purchased from Life Technologies (San Giuliano Milanese, Italy); lipopolysaccaride (LPS) (Ultra-Pure LPS-EB from *E. coli* 0111:B4 strain; only activates TLR4), Pam_3_CSK_4_, and ethyl-(6R)-6-(N-(2-chloro-4-fluorophenyl)sulfamoyl)cyclohex-1-ene-1-carboxylate (CLI-095 or TAK 242) were from InvivoGen (Cayla-Invivogen Europe, Toulouse, France); A740003 from Tocris Bioscience, Pittsburgh, PA, USA); poly-L-lysine hydrobromide (mol wt 70,000–150,000), papain, DNase I (bovine pancreas), trypsin inhibitor, L-leucyl-L-leucine methyl ester (L-LME), protease inhibitor cocktail, Pefabloc® SC (100 mM), CU-CPT22, and all other biochemicals were purchased from Sigma-Aldrich (Milan, Italy) unless noted otherwise; recombinant human Apo-SAA (consensus SAA molecule corresponding to human Apo-SAA1α, except for the presence of an N-terminal methionine, the substitution of asparagine for aspartic acid at position 60, and arginine for histidine at position 71) from Peprotech (endotoxin level < 0.1 ng/μg protein; London, UK); QIAzol from Qiagen (Milan, Italy. Falcon tissue culture plastic-ware were purchased from BD Biosciences. Sterilin Petri plastic dishes (10 cm Ø) were obtained from Sarstedt (Verona, Italy).

### Cell culture

Microglia were isolated from mixed glial cell cultures as previously described [34]. All experiments were conducted in compliance with Italian Ministry of Health (art. 31, D.L. 26/2014) guidelines for the care and use of laboratory animals and were approved by the Institutional Animal Care and Use Committee of the University of Padua (958/2016-PR). In brief, cells dissociated from postnatal day 1 rat pups (Charles River, Calco, Italy; strain: CD) cerebral cortices were plated in 75 cm^2^ poly-L-lysine-coated tissue culture flasks (1.5 brains per flask) and grown in high-glucose Dulbecco’s modified Eagle’s medium (DMEM) with 2 mM glutamine, 50 units/ml penicillin/50 μg/ml streptomycin, 50 μg/ml gentamycin, and 10% FCS (‘growth medium’). Culture medium was changed after 24 h. The cultures reached confluence by 7 days at which time microglia were recovered by shaking the flasks on an orbital shaker at 200 rpm for 1 h (37 °C). The remaining cell monolayers were highly enriched in astrocytes (< 5% microglia, as determined by flow cytometry using cell type-specific antibodies) [35]. The culture supernatant containing microglia was transferred to Sterilin plastic Petri dishes and incubated for 45 min at 37 °C (5% CO_2_, 95% air) to allow adhesion of microglia. The adherent microglial cells (> 99% pure, as determined by flow cytometry using cell type-specific antibodies) [35] were detached by mechanically scraping into growth medium and re-plated in this same medium, on poly-L-lysine-coated 24-well or 96-well culture plates (250,000 and 50,000 cells per well for mRNA and cytokine analysis, respectively). For some experiments, the astrocyte monolayers were depleted of residual microglia using a 60-min exposure (50 mM) to the lysosomotropic agent L-LME [36], as described previously [37, 38]. Astrocyte plating densities were the same as used for microglia.

### Quantitative real-time polymerase chain reaction (q-PCR)

Total RNA was extracted from cells by QIAzol, according to the manufacturer’s instructions. RNA integrity and quantity were determined by RNA 6000 Nano assay in an Agilent BioAnalyser (A_260/280_ ratio > 1.8). Reverse transcription was performed with Superscript III reverse transcriptase (Invitrogen). The q-PCR reaction was performed as described previously [37]. Primer sequences are listed in Table 1. Amounts of each gene product were calculated using linear regression analysis from standard curves, demonstrating amplification efficiencies ranging from 90 to 100%. Dissociation curves were generated for each primer pair, showing single product amplification. Data are normalized to β-actin mRNA level.

**Table 1:** PCR primers used in this study

Gene target	Direction	Sequence
β-ACT	F	5′-CCCCATTGAACACGGCATTGTCA-3′
	R	5′-ACCCTCATAGATGGGCACAGTGT-3′
IL-1β	F	5′-TGTGGCAGCTACCTATGTCT-3′
	R	5′-GGGAACATCACACACTAGCA-3′
NLRP3	F	5′-TGATGCATGCACGTCTAATCTC-3′
	R	5′-CAAATCGAGATGCGGGAGAG-3′
SAA1	F	5′-ACACGGAGCAGAGGACTCAAG-3′
	R	5′-GGTCGAAAGTGGTTGGGGTC-3′
TNF-α	F	5′-CATCTTCTCAAAACTCGAGTGACAA-3′
	R	5′-TGGGAGTAGATAAGGTACAGCCC-3′
TLR2	F	5′-TCCATGTCCTGGTTGACTGG-3′
	R	5′-AGGAGAAGGGCACAGCAGAC-3′
TLR4	F	5′-GATTGCTCAGACATGGCAGTTTC-3′
	R	5′-CACTCGAGGTAGGTGTTTCTGCTAA-3′

β*-ACT* β-actin, *SAA* serum amyloid A, *IL-1β* intereukin-1β, *TNF-α* tumor necrosis factor α, *NLRP3* inflammasome, *TLR* toll-like receptor. *F* forward, *R* reverse

### IL-1β production and release

Purified microglia and enriched astrocytes were plated in poly-L-lysine coated 96-well plates (50,000 cells per well) in growth medium and allowed to adhere overnight. These plating densities do not affect glial cell vitality/function [39, 40]. Cells were primed by pre-treating with 0.1 μg/ml LPS (optimal concentration chosen from preliminary experiments) [18, 37, 41] or different concentrations of recombinant human Apo-SAA (as indicated in the experiment) for 2 h in serum-free culture medium prior to stimulation with 5 mM ATP [41] for 1 h. None of the treatments, at the concentrations tested, affected cell viability (data not shown; see also [12, 42–44]). Cell supernatants were collected and stored at − 20 °C until the day of assay (avoiding repeated freeze-thaw cycles). Cell lysates were prepared by adding to each 96-well culture 100 μl lysis solution containing: 89 μl NP40 lysis buffer, 10 μl of 10× protease inhibitor cocktail, and 1 μl of 100 mM Pefabloc SC. IL-1β content of culture medium and cell lysates was analyzed using commercially available enzyme-linked immunosorbent assay (ELISA) kits according to the manufacturer’s instructions (Antigenix America, Huntington Station, NY, USA). Standards with known amounts of IL-1β and TNF-α were used to convert values into absolute concentrations of the cytokine in pg/ml.

### Statistics

Data are given as mean ± sem, unless stated otherwise. Statistical analyses to determine group differences were performed either by two-sample equal variance Student’s *t* test, or by one-way analysis of variance followed by Dunnett’s or Bonferroni’s post hoc tests for comparisons involving more than two data groups. Significance was taken at *p* < 0.05.

## Results

### SAA upregulates NLRP3 and IL-1β mRNA in rat primary microglia

**Fig. 1:**
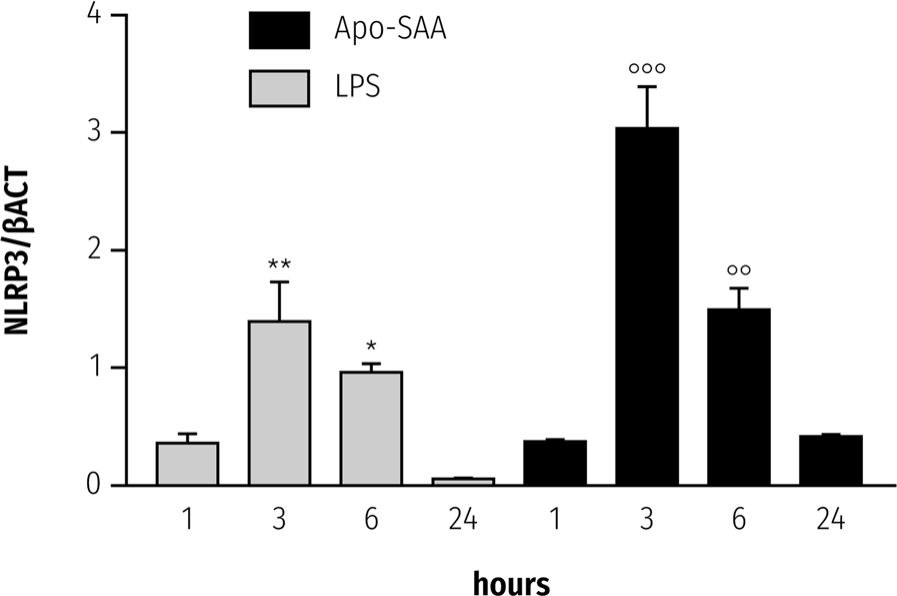
Treatment of rat cortical microglia with Apo-SAA or LPS upregulates in a time-dependent manner mRNA for NLRP3. Cultures were treated the day after plating with 0.5 μg/ml recombinant human Apo-SAA or 0.1 μg/ml LPS and processed 1, 3, 6, and 24 h later for q-PCR, as detailed in the “Methods” section. Data are presented as relative expression level (normalized with respect to β-actin (βACT)) at each time point and are mean ± sem, *n* = 6. Control values (which were at the limit of detection) were omitted for clarity. Apo-SAA (■); LPS (▓). For LPS, *p < 0.05 and **p < 0.01 vs 1 h; for Apo-SAA, **°°***p* < 0.01 and **°°°***p* < 0.001 vs 1 h

**Fig. 2:**
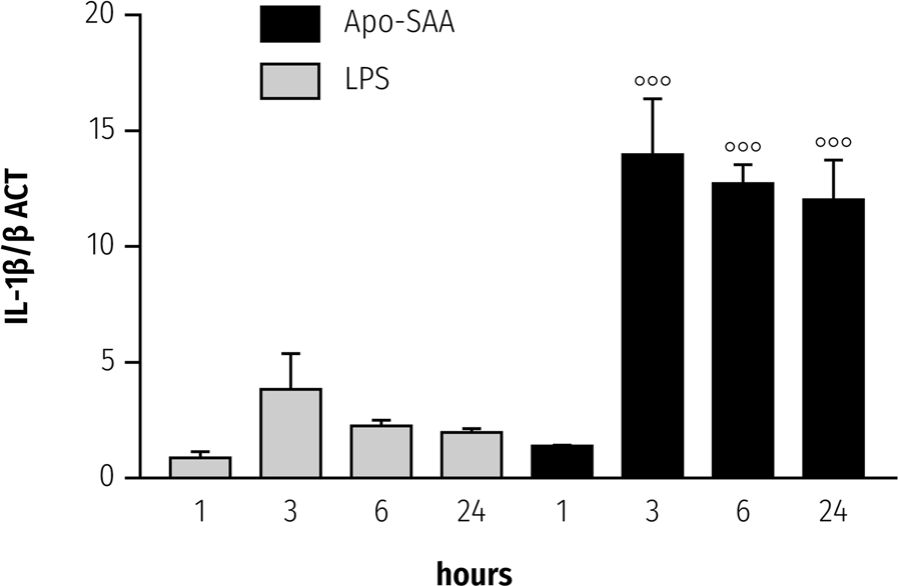
Treatment of rat cortical microglia with Apo-SAA or LPS upregulates in a time-dependent manner mRNA for IL-1β. Cultures were treated the day after plating with 0.5 μg/ml recombinant human Apo-SAA or 0.1 µg/ml LPS and processed 1, 3, 6, and 24 h later for q-PCR, as detailed in the “Methods” section. Data are presented as relative expression level (normalized with respect to β-actin (βACT)) at each time point and are mean ± sem, *n* = 3. Control values (which were at the limit of detection) were omitted for clarity. Apo-SAA (■); LPS (▓). For Apo-SAA, **°°°***p* < 0.001 vs 1 h

**Fig. 3:**
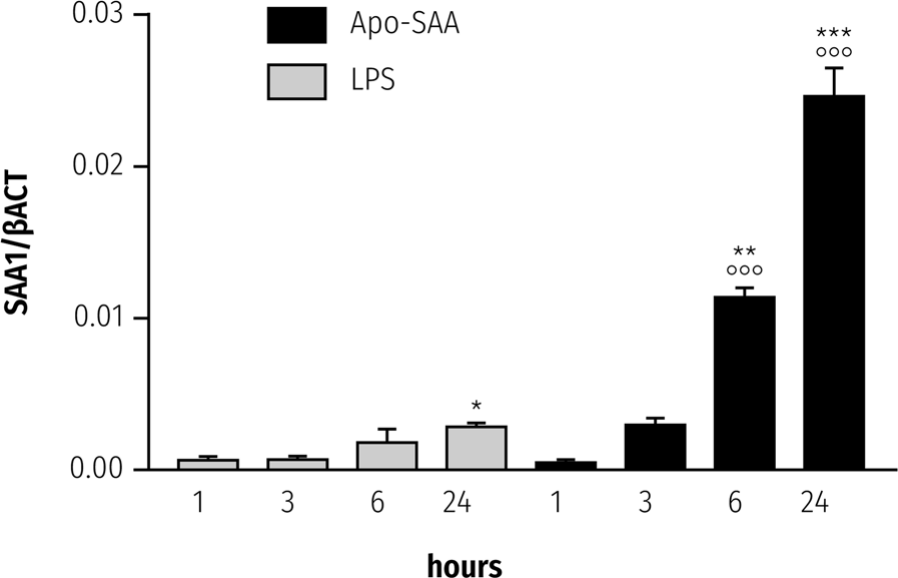
Treatment of rat cortical microglia with Apo-SAA or LPS upregulates in a time-dependent manner mRNA for SAA1. Cultures were treated the day after plating with 0.5 μg/ml recombinant human Apo-SAA or 0.1 µg/ml LPS and processed 1, 3, 6, and 24 h later for q-PCR, as detailed in the “Methods” section. Data are presented as relative expression level (normalized with respect to β-actin (βACT)) at each time point and are mean ± sem, *n* = 6. Control values (which were at the limit of detection) were omitted for clarity. Apo-SAA (■); LPS (▓). For LPS, *p < 0.05 vs 1 h; for Apo-SAA, **°°°***p* < 0.001 vs 1 h, **p < 0.01 vs 1 h, and ***p < 0.001 vs 6 h

SAA mediates cytokine production by a variety of non-neural cell types [45], as well as primary microglia [12]. However, it is not known if SAA is able also to upregulate expression of the NLRP3 inflammasome gene in CNS-derived glia. Incubation of rat primary cortical microglia with Apo-SAA resulted in a time-dependent rise in *Nlrp3* expression that peaked at 3 h (Fig. 1, solid bars); a similar time-course was observed when microglia were stimulated with LPS (Fig. 1, shaded bars). Relative expression levels appeared higher for Apo-SAA than for LPS-treated cells (optimal concentrations chosen for Apo-SAA and LPS were based on preliminary experiments; see also [12, 35]). The kinetics of *Tnf-α* expression in Apo-SAA- and LPS-stimulated microglia mirrored that seen for NLRP3 mRNA (Additional file 1: Figure S1). Interestingly, the expression level of *Il-1β* in both Apo-SAA- and LPS-treated cells peaked already at 3 h (Fig. 2, solid and shaded bars, respectively) and remained at plateau through 24 h for Apo-SAA. In contrast, intracellular content of IL-1β peaked at 6 h in both Apo-SAA- and LPS-treated microglia and then declined, although remained above basal up to 24 h (Additional file 2: Figure S2). Exposure of microglia to either Apo-SAA or LPS led to a gradual up-regulation of *Saa1* over the 24-h study period (Fig. 3, solid and shaded bars, respectively), suggesting a potential autocrine/paracrine effect of SAA. Again, the effect of Apo-SAA was more robust than that of LPS in terms of relative expression levels for *Saa1*.

### SAA primes cortical and cerebellar microglia for ATP- and P2X_7_R-dependent IL-1β release

**Fig. 4:**
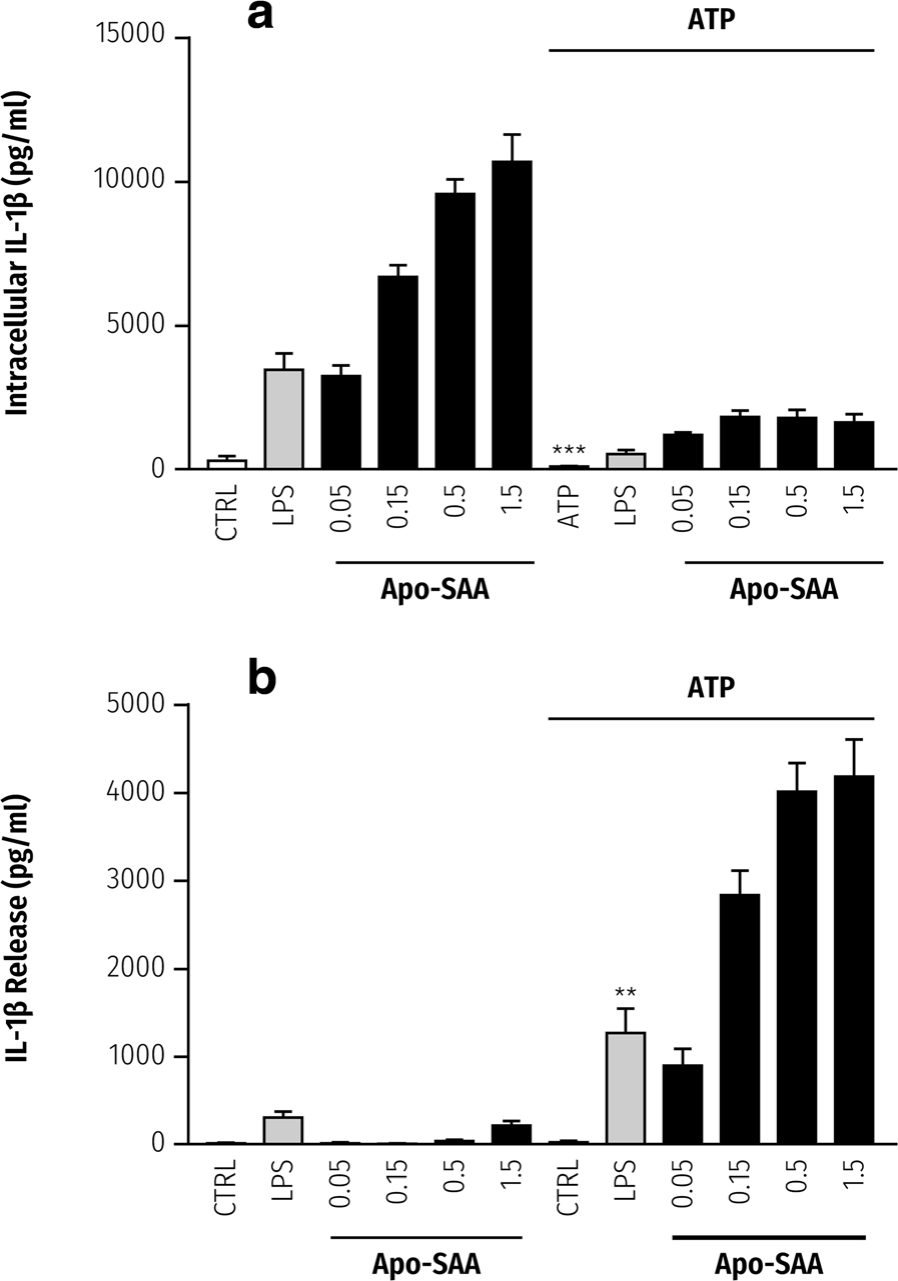
Extracellular ATP causes release of IL-1β from rat cortical microglia primed with Apo-SAA or LPS. Cortical microglia cultured in 96-well plates were first incubated 2 h with 0.05–1.5 μg/ml recombinant human Apo-SAA or 0.1 μg/ml LPS. ATP was then added to a final concentration of 5 mM as indicated by upper horizontal bar. After a further 60-min incubation, cell lysates (**a**) and culture medium (**b**) were collected for IL-1β analysis by ELISA. Note that Apo-SAA was again a more efficacious priming stimulus than was LPS. Control (CTRL). Apo-SAA (■); LPS (▓). Data are mean ± sem (*n* = 6). **a** **p < 0.01 vs LPS only (no ATP). Apo-SAA at all concentrations differed significantly with added ATP (*p* < 0.001) compared to the same concentration with no ATP. **b** ***p < 0.001 vs LPS only (no ATP). Apo-SAA at all concentrations differed significantly with added ATP (*p* < 0.001) compared to the same concentration with no ATP

In the brain, IL-1β is mainly produced by activated microglia [46], and ATP-dependent/P2X_7_R-triggered IL-1β maturation and export is believed to contribute significantly to this cytokine pool in nervous system pathologies [20, 23]. TLR4/TLR2 signaling pathways may be involved in neurodegenerative disorders including motor neuron disease [47], cerebral hypoxia-ischemia [48, 49] and blood-spinal cord barrier dysfunction after ischemia/reperfusion injury [50], and neuropathic pain [51]. Using a protocol established for TLR agonist priming of primary CNS glia [38], cortical microglia were first incubated with different concentrations of Apo-SAA for 2 h in serum-free medium followed by addition of ATP to a final concentration of 5 mM. After a further 60 min of incubation, cell culture medium and cell lysates were collected for measurement of IL-1β by ELISA. Incubation with Apo-SAA only (no ATP) led to a concentration-dependent accumulation of intracellular IL-1β that was already maximal at 0.5 μg/ml Apo-SAA, with very little IL-1β in the culture medium (left half of Fig. 4a, b, respectively). Conversely, incubation with Apo-SAA/ATP produced a concentration-dependent accumulation of IL-1β in the culture medium (again maximal at 0.5 μg/ml Apo-SAA), with a concomitant loss of intracellular IL-1β (right half of Fig. 4a and b, respectively, solid bars). Qualitatively similar effects were seen with 0.1 μg/ml LPS (maximal effective concentration), although once again the magnitude of cytokine production was greater for Apo-SAA.

Routinely used methods for preparing rodent primary astrocyte cell cultures generally contain variable, small percentages (up to 5%) of contaminating microglia [52]. A number of studies have demonstrated that inflammatory mediator output from enriched astrocytes is dependent on the presence of residual microglia [12, 37, 38, 53, 54]. Utilizing the lysosomotropic agent L-LME [55] to remove any remaining microglia [36–38, 56–58], purified cortical astrocytes were unresponsive to priming by Apo-SAA, as observed earlier when using LPS [38] (data not shown).

**Fig. 5:**
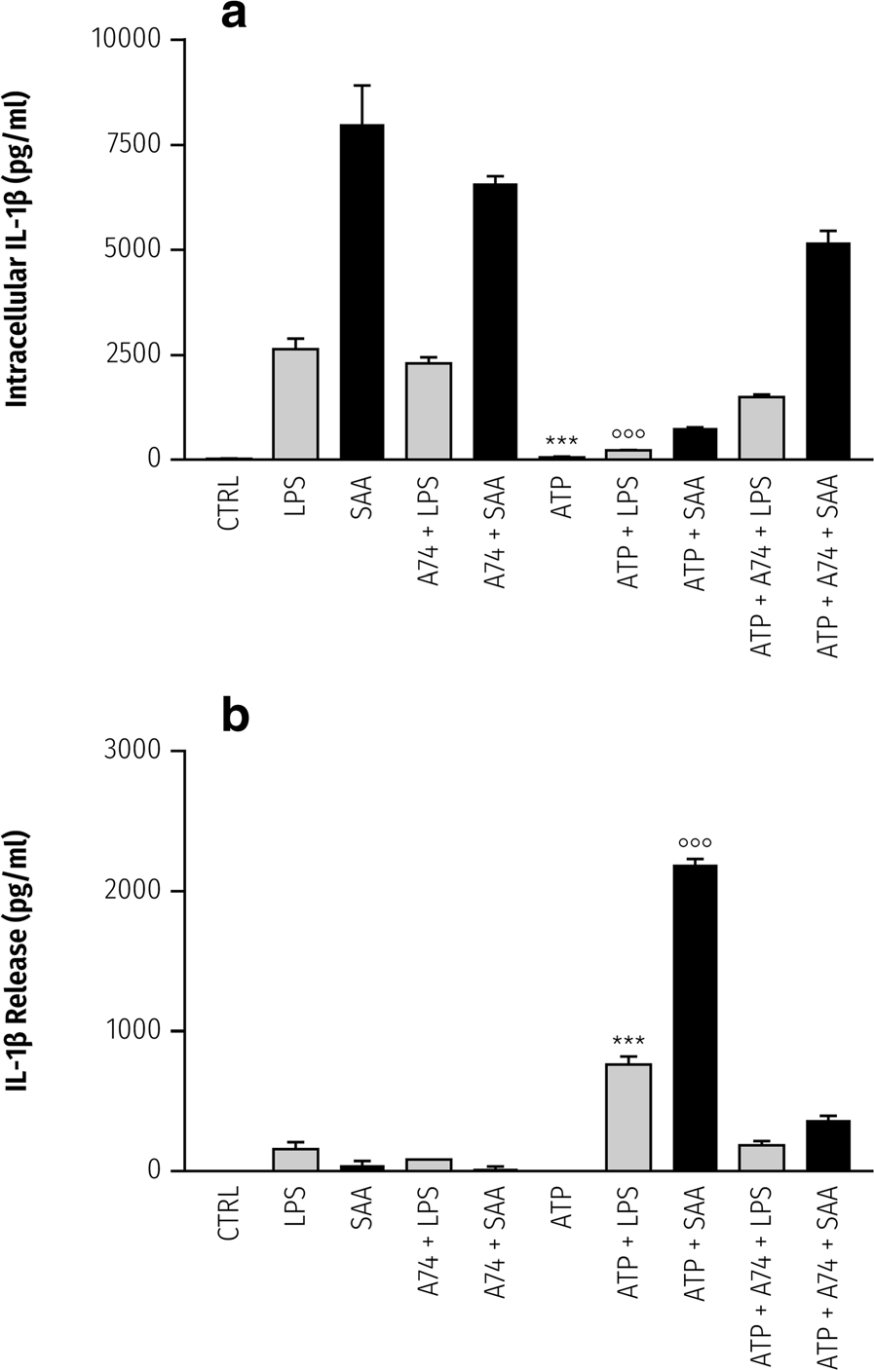
The P2X_7_R antagonist A740003 prevents the ATP-dependent fall in intracellular IL-1β (**a**) and rise in extracellular IL-1β (**b**) in Apo-SAA- or LPS-primed rat cortical microglia. Microglia cultured in 96-well plates were pretreated for 30 min with 10 μM A740003 (‘A74’) followed by a 2-h incubation with 0.5 μg/ml recombinant human Apo-SAA (‘SAA’) or 0.1 μg/ml LPS. ATP was then added to a final concentration of 5 mM. After a further 60-min incubation, culture medium (**b**) and cell lysates (**a**) were collected for IL-1β analysis by ELISA. Apo-SAA (▓); LPS (▄) Data are mean ± sem (*n* = 3). ***p < 0.001 vs LPS or LPS + ATP + A740003; **°°°***p* < 0.001 vs Apo-SAA or Apo-SAA + ATP + A740003. There were no statistically significant differences between the LPS/LPS + A740003/LPS + ATP + A740003 and Apo-SAA/Apo-SAA + A740003/Apo-SAA + ATP + A740003 groups

To interrogate a role for P2X_7_R in the Apo-SAA/ATP-mediated IL-1β release, microglia were pretreated 30 min with the selective P2X_7_R antagonist A740003 [59], then primed 2 h with Apo-SAA followed by a further 1-h incubation with ATP. The expected rise in intracellular IL-1β caused by exposure to either Apo-SAA or LPS was unaffected by A740003, while the fall in intracellular IL-1β in cells treated with Apo-SAA or LPS and ATP did not occur (Fig. 5a). In contrast, A740003 prevented the increase in extracellular IL-1β in Apo-SAA- or LPS-primed rat cortical microglia treated with ATP (Fig. 5b). These observations most likely are a consequence of blocking P2X_7_R-dependent cleavage of IL-1β precursor to the releasable mature form of the cytokine.

Purified microglia cultured from rat cerebellum also respond to ATP-dependent IL-1β release upon priming with TLR2, TLR3, and TLR4 isoform agonists, in a P2X_7_R-dependent manner [38].

**Fig. 6:**
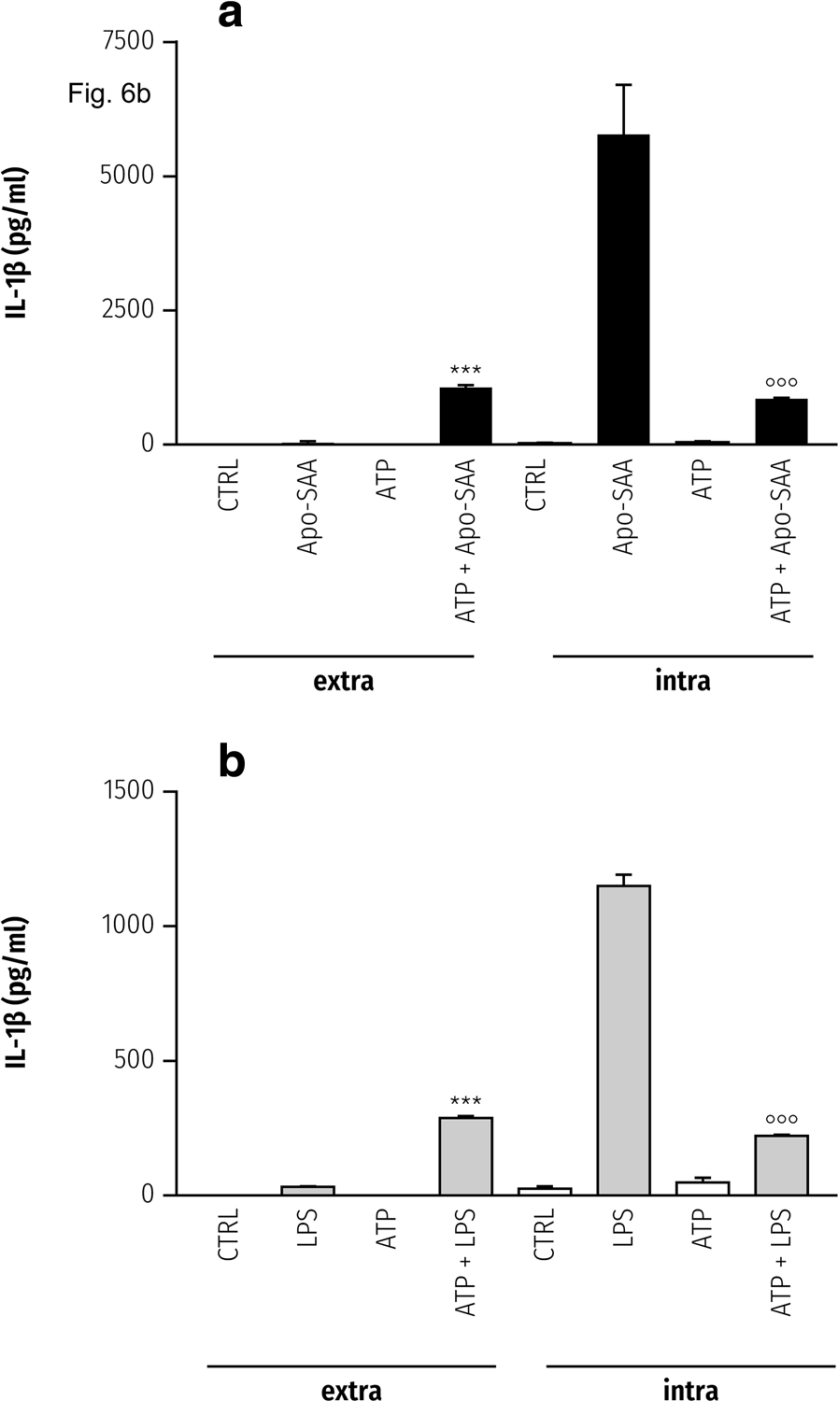
Extracellular ATP causes release of IL-1β from purified rat cerebellar microglia primed with Apo-SAA or LPS. Microglia cultured in 96-well plates were first incubated 2 h with 0.5 μg/ml recombinant human Apo-SAA (**a**) or 0.1 μg/ml LPS (**b**). ATP was then added to a final concentration of 5 mM. After a further 60-min incubation, culture medium (‘extra’) and cell lysates (‘intra’) were collected for IL-1β analysis by ELISA. As for cortical microglia, here also Apo-SAA was a more efficacious stimulus than was LPS (note the difference in y-axis scales). Control (CTRL). Data are mean ± sem (*n* = 6). ***p < 0.001: Apo-SAA vs Apo-SAA + ATP; or LPS vs LPS + ATP (extracellular). **°°°***p* < 0.001 vs Apo-SAA vs Apo-SAA + ATP; or LPS vs LPS + ATP (intracellular)

To determine if this is also the case for priming with Apo-SAA, cerebellar microglia were incubated for 2 h with 0.5 μg/ml recombinant human Apo-SAA followed by addition of ATP to 5 mM or culture medium only. As Fig. 6a shows, Apo-SAA caused a clear rise in the intracellular content of IL-1β, which was markedly diminished in the presence of ATP. Conversely, IL-1β in the culture medium increased for Apo-SAA + ATP-treated cells. Qualitatively analogous results were obtained using LPS (Fig. 6b), as seen previously [38].

### Apo-SAA modulates TLR2 and TLR4 gene expression in cortical microglia

**Fig. 7:**
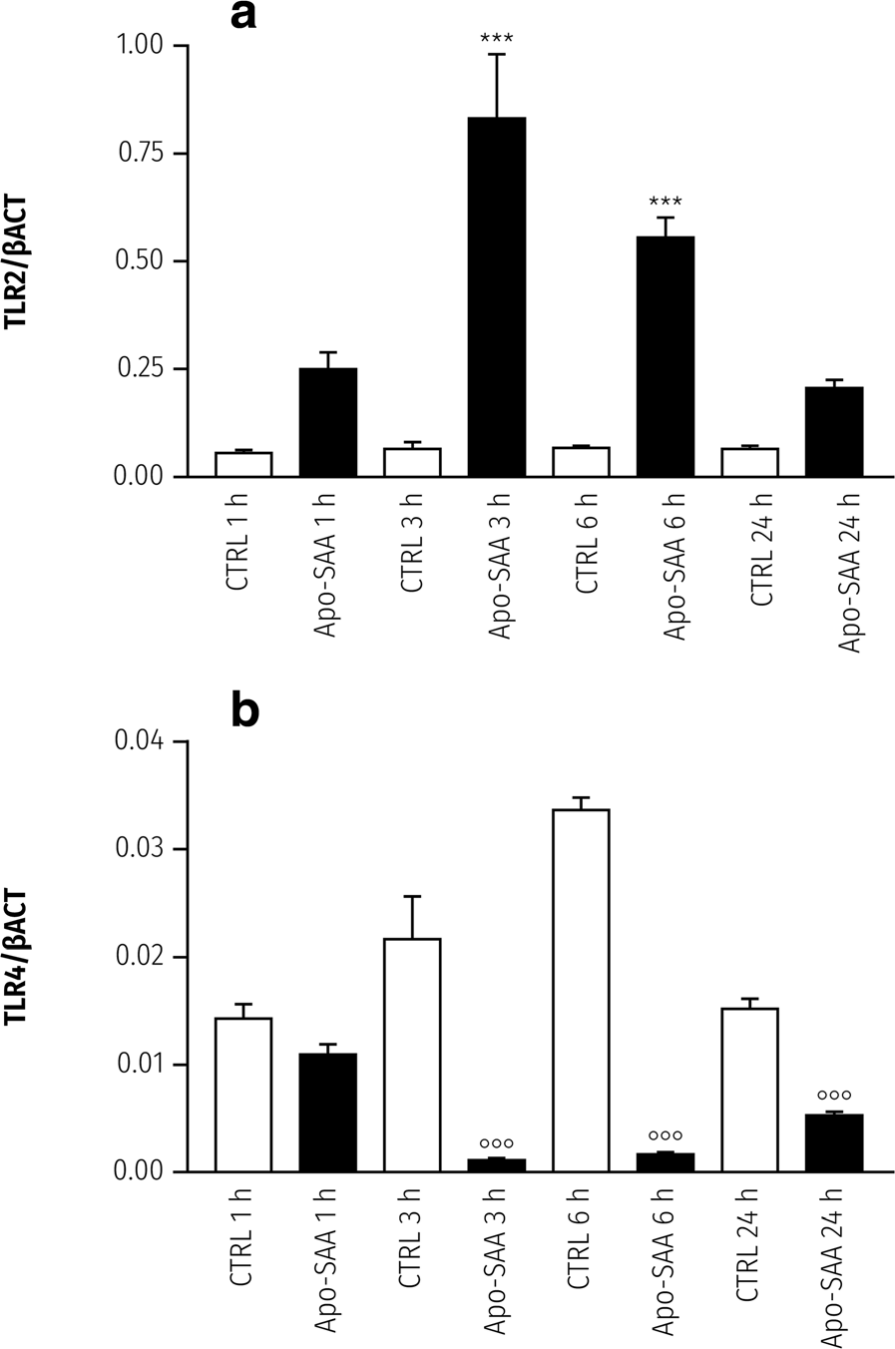
Treatment of rat cortical microglia with Apo-SAA, in a time-dependent manner, upregulates mRNA for TLR2 and downregulates that for TLR4. Cultures were treated the day after plating with 0.5 μg/ml recombinant human Apo-SAA and processed 1, 3, 6, and 24 h later for q-PCR, as detailed in the “Methods” section. **a** TLR2. **b** TLR4. Data are presented as relative expression level (normalized with respect to β-actin (βACT)) at each time point and are mean ± sem, *n* = 3 (*n* = 9 for 3 h). Control (CTRL; untreated) cultures. ***p < 0.001 vs CTRL for that time point; **°°°***p* < 0.001 vs CTRL for that time point. Note the difference in expression levels between TLR2 and TLR4 mRNA

In purified primary rat cortical microglia, TLR4 and TLR2 agonists have been reported to upregulate TLR2 mRNA expression while downregulating that of TLR4 [35]. Given that SAA is a putative agonist for both TLR4 and TLR2, we asked whether SAA could also affect *Tlr2* and *Tlr4* expression in cortical microglia. Apo-SAA treatment of microglia time-dependently increased *Tlr2*, with a peak at 3 h and a decline over 24 h (Fig. 7a). In contrast, *Tlr4* expression (normalized to β-actin) was significantly reduced at 3, 6, and 24 h (Fig. 7b). In confirmation of previous findings [35], LPS also raised *Tlr2* expression at 3 h, while downregulating that of *Tlr4* eat all time points (Additional file 3: Figure S3).

Several studies suggest that SAA is capable of activating TLR4 [24–27] and TLR2 [28–31]. To explore this in the present setting, cortical microglia were first pre-incubated for 30 min with either the TLR4 antagonist CLI-095 [60] or the TLR2 antagonist CU-CPT22 [61]. The former (also known as TAK-242) suppresses specifically TLR4 signaling, inhibiting the production of pro-inflammatory cytokines [62]. CLI-095 acts by blocking signaling mediated by the intracellular domain of TLR4, but not the extracellular domain, and suppresses both ligand-dependent and ligand-independent signaling of TLR4 [62]. CU-CPT22 is reported to compete with the synthetic triacylated lipoprotein (Pam_3_CSK_4_) binding to TLR1/2 and repress downstream signaling from IL-1β and TNF-α [61].

**Fig. 8:**
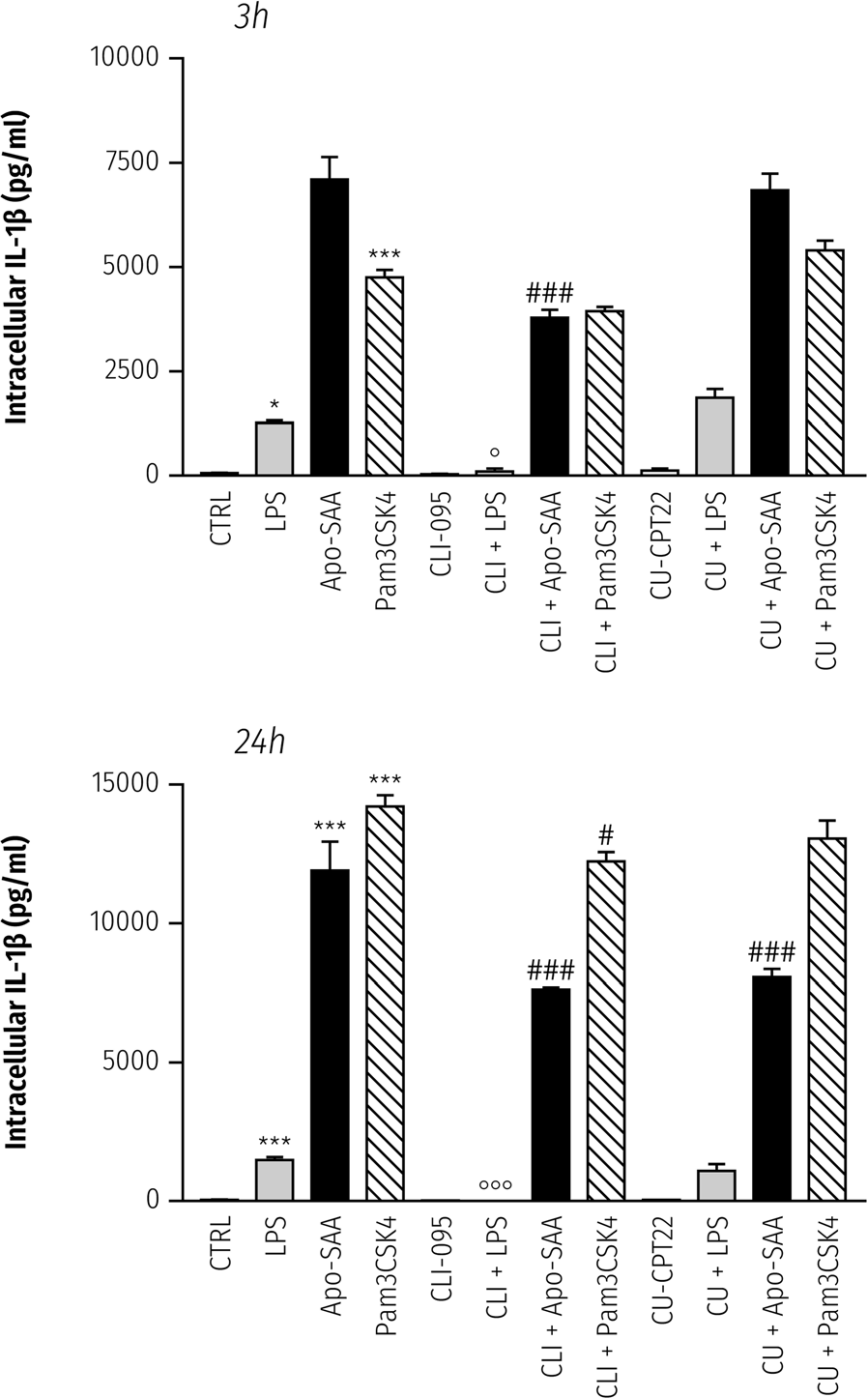
Effect of the TLR4 antagonist CLI-095 and the TLR1/2 antagonist CU-CPT22 on intracellular IL-1β content in rat cortical microglia treated with LPS, Apo-SAA, or Pam_3_CSK_4_. The day after plating culture medium was replaced with 50 μl/well of serum-free medium containing 1 μg/ml CLI-095 (‘CLI’) or 20 μM CU-CPT (‘CU’). After 30-min incubation, equal volume of medium was added containing 0.2 μg/ml LPS, 1 μg/ml recombinant human Apo-SAA, or 0.6 μg/ml Pam_3_CSK_4_. Final concentrations of all added agents were thus one-half of the values indicated. After a further incubation of 3 and 24 h cell lysates were collected for IL-1β analysis by ELISA, as detailed in the “Methods” section. (upper panel) 3 h; (lower panel) 24 h. Data are mean ± sem, *n* = 4. Control (CTRL; untreated) cultures. *p < 0.05 and ***p < 0.001 vs CTRL; **°***p* < 0.05 and **°°°***p* < 0.001 vs LPS; ^**###**^*p* < 0.001 vs Apo-SAA; ^**#**^*p* < 0.05 and vs Pam_3_CSK_4_

Subsequent addition of LPS (0.1 μg/ml), Apo-SAA (0.5 μg/ml), or the TLR2 agonist Pam_3_CSK_4_ (0.3 μg/ml) produced significant increases in the intracellular content of IL-1β after both 3 and 24 h (Fig. 8, upper and lower panels, respectively). CLI-095 pre-treatment fully blocked the effect of LPS, and partially (but significantly, *p* < 0.001) that of Apo-SAA at both time points. However, the stimulatory effect of Pam_3_CSK_4_ was unaffected by the TLR4 antagonist at 3 h, and modestly so at 24 h. CU-CPT22 failed to alter the stimulatory effect of Apo-SAA or LPS; surprisingly, this TLR1/2 antagonist also was ineffective when tested on cells challenged with the TLR1/2 agonist Pam_3_CSK_4_—in contrast to earlier findings [61]. Similar results were obtained for TNF-α release from the same cells (data (pg/ml) are mean ± sem, 3-h point): control, CLI-095, and CU-CPT22-treated cells, 0; LPS, 311 ± 24; Apo-SAA, 840 ± 102; Pam_3_CSK_4_, 322 ± 9; LPS + CLI-095, 0; Apo-SAA + CLI-095, 224 ± 3; Pam_3_CSK_4_ + CLI-095, 242 ± 12; LPS + CU-CPT22, 399 ± 23; Apo-SAA + CU-CPT22, 822 ± 76; 420 ± 3; CLI-095 fully eliminated the effect of LPS and partially, but significantly, that of Apo-SAA (*p* < 0.001).

## Discussion

Growing evidence indicates that CNS disorders are characterized by central activation of innate immunity, as well as activation of a potent peripheral acute phase response that influences central inflammation and leads to poor disease outcome [5]. The acute phase response plays a critical role in the innate immune response to tissue injury [63]. Among acute phase proteins such as C-reactive protein, complement proteins, and SAA, the last one can be considered a “danger signal” that influences the inflammation process [64]. Its low basal level and high inducibility are in keeping with danger signal molecules [65], being produced in response to potentially harmful environmental cues, including trauma, infection, surgery, and severe stress. A number of studies imply a role for SAA in inflammation-associated neuropathologies [7–11], although the underlying molecular processes remain to be fully explored. Here, we show that in neonatal cortical microglia, Apo-SAA time-dependently upregulates NLRP3 inflammasome and IL-1β mRNA expression and intracellular production of IL-1β and stimulates release of IL-1β in the presence of ATP, a multi-target danger signal in the brain [32] in a P2X_7_R-dependent manner. The rise in extracellular release of IL-1β in the presence of ATP was accompanied by a fall in the intracellular content, consistent with NLRP3/caspase 1-complex 1 activation and cleavage of the pro-form of IL-1β to the mature, active secreted species [20–22]. The action of Apo-SAA was not limited to cortical microglia, as similar ATP-dependent release of IL-1β was also seen for cerebellar microglia. IL-1β is viewed as a master regulator of neuroinflammation [13] that contributes importantly to cellular activation and cytokine production. This cytokine plays a key role in the pathogenesis of acute and chronic diseases of both the peripheral nervous system and CNS [15–17]. It merits mention that the effects of Apo-SAA on gene expression and cytokine production were of a far greater magnitude than those obtained using the optimal concentration of LPS as benchmark, thus highlighting the pro-inflammatory potency of this acute phase protein. Whether adult microglia would respond differently was not tested.

A comparison of the SAA concentrations used in the present investigation with levels of SAA previously detected in human cerebrospinal fluid and plasma suggests the potential for physiological relevance to the in vivo setting. In one report, SAA levels in cerebrospinal fluid of AD subjects were found to be much higher than in normal controls [8], and generally within the range of the highest concentration used here. Serum concentrations of SAA in relapsing-remitting MS patients have been reported elevated with a mean level of 12.1 ± 8.7 μg/ml [66], and significantly increased (mean value 10 μg/ml, *p* = 0.030 vs. control) in neuromyelitis optica patients [67]. These values contrast with active concentrations of 0.15–1.5 μg/ml in the present in vitro study.

An expanding body of data demonstrates that pharmacological or genetic manipulation of P2X_7_Rs alters their responsiveness in animal models of CNS neurological disorders [33, 68]. The P2X_7_R has been suggested to also regulate the pathophysiology of psychiatric disorders, including mood disorders [33]. P2X_7_R-triggered IL-1β maturation and export is thus likely to represent an important contributor to this cytokine pool [20, 23]. SAA is not detectable in normal brain but has been reported in AD brain, together with its gene in MS brain [7]. Miida et al. [8] described a raised SAA concentration in cerebrospinal fluid of AD. SAA immunoreactivity was reported to co-localize with amyloid β-peptide deposits in AD brain [9]. P2X_7_R-positive microglia surrounded amyloid plaques in a mouse transgenic AD model [69], and microglia around amyloid plaques in AD brain are immunopositive for IL-1β [70]. Collectively, these findings propose a link between P2X_7_R, Apo-SAA, and IL-1β in AD pathophysiology. In addition, the ability of Apo-SAA to regulate its own gene expression suggests the potential for autocrine/paracrine effects of SAA. Since microglia in the AD brain adopt distinct functional and molecular phenotypes, it is conceivable that the response of “AD microglia” to SAA would differ from that of wild-type microglia.

A number of reports indicate the capability of SAA to act as an agonist for both TLR4 [24–27] and TLR2 [28–31, 71]. Ligand engagement of TLR4 by LPS and TLR2 by Pam_3_CSK_4_ leads to the upregulation of *Tlr2* and downregulation of *Tlr4* in cortical microglia [35]. Consistent with its putative action as a ligand for both TLR2 and TLR4, Apo-SAA produced a time-dependent robust and significant increase in *Tlr2* expression in cortical microglia, with a concomitant reduction in the relative level of *Tlr4*. Conceivably, this action of SAA could result in a ‘feed-forward’ mechanism, whereby SAA increases expression of its receptor and amplification of a priming response. Attempts at using pharmacological tools to dissect participation of TLR4 and TLR2 in the actions of Apo-SAA were equivocal. The selective TLR4 antagonist CLI-095 completed blocked the ability of LPS to synthesize/release IL-1β and partially, but significantly, that of Apo-SAA. While the TLR2 antagonist CU-CPT22 failed to alter the stimulatory effect of Apo-SAA or LPS, it also proved ineffective on microglia treated with the TLR1/2 agonist Pam_3_CSK_4_. Our inability to confirm the earlier report for CU-CPT22 action against Pam_3_CSK_4_ [61] could be due to differences in cell type used (primary microglia vs RAW264.7 macrophages) or treatment times (not specified in [61]), even though we used two incubation times and the same concentrations of ligand and antagonist as in [61]. Another consideration is that CU-CPT22 was designed to compete with Pam_3_CSK_4_ binding to TLR1/2, thus disrupting formation of the TLR1/TLR2 heterodimer [61]. Although outside the scope of the present study, the use of microglia from TLR2^**−/−**^ animals could provide a tool to address this question.

A failure of remyelination is responsible, in large part, for the long-term neurologic consequences of MS. An intriguing study by Sloane et al. [72] described upregulated TLR2 expression by oligodendrocytes in MS lesions, with pathogen-derived TLR2 agonists, but not agonists for other TLRs, inhibiting oligodendrocyte precursor cell (OPC) maturation in vitro. Ablated expression of TLR2 also enhanced remyelination in a lysolecithin animal model of MS [72]. Intense immunohistochemical staining of SAA has been detected in the brains of patients with neurologically confirmed MS in comparison to an unaffected region and non-MS brains, with the major site of staining being the myelin sheaths of axons in affected cortex [11]. Pro-inflammatory cytokines such as IL-1β and TNF-α [73] may play important roles in expression of SAA1 and SAA2. The pathophysiology of a variety of neurological disorders, including MS, is associated with TNF-α [74, 75], a master pro-inflammatory product of activated microglia and peripheral macrophages implicated in the pathogenesis of CNS demyelination [76, 77]. Apo-SAA treatment of rat cortical microglia increased production of TNF-α and IL-1β, while TNF-α time-dependently raised *Saa1* expression in cultured OPCs [12]. Our findings in the context of the above considerations, together with evidence for P2X_7_R in the development of experimental autoimmune encephalomyelitis [78] and microglia-oligodendrocyte crosstalk [79], propose a vicious cycle of Apo-SAA, IL-1β, and TLR2 leading to the demise of OPCs.

## Conclusions

CNS disorders are characterized by central activation of innate immunity and activation of a potent peripheral acute phase response that influences central inflammation and contributes to poor outcome. Our data show that in microglia, the acute phase protein Apo-SAA upregulates NLRP3 inflammasome and IL-1β mRNA expression and intracellular production of IL-1β and stimulates release of IL-1β in the presence of ATP in a P2X_7_R-dependent manner. Apo-SAA upregulated expression of its own gene and that of TLR2, suggesting a potential ‘feed-forward’ mechanism, whereby SAA increases expression of its receptor and amplification of a priming response. The effects of Apo-SAA on gene expression and cytokine production were of a far greater magnitude than those observed with the classical TLR4 agonist lipopolysaccharide, highlighting the pro-inflammatory potency of this acute phase protein. Given the evidence for P2X_7_Rs involvement in CNS neurological disorders and expression of SAA in AD and MS brain, the findings presented here propose a link between P2X_7_R, SAA, and IL-1β in CNS pathophysiology.

## Additional files

Additional file 1: **Figure S1.** Treatment of rat cortical microglia with Apo-SAA or LPS upregulates, in a time-dependent manner mRNA for TNF-α. Cultures were treated the day after plating with 0.5 μg/ml recombinant human Apo-SAA or 0.1 µg/ml LPS and processed 1, 3, 6, and 24 h later for q-PCR, as detailed in the “Methods” section. Data are presented as relative expression level (normalized with respect to β-actin (βACT)) at each time point and are mean ± sem, *n* = 3. Control values (which were at the limit of detection) were omitted for clarity. Apo-SAA (■); LPS (▓). For LPS, *p < 0.05 vs 1 and 24 h; for Apo-SAA: **°°°***p* < 0.001 vs 1, 6, and 24 h. (TIF 468 kb)

Additional file 2: **Figure S2.** Treatment of rat cortical microglia with Apo-SAA or LPS upregulates, in a time-dependent manner, intracellular content of IL-1β. Cultures were treated the day after plating with 0.5 μg/ml recombinant human Apo-SAA or 0.1 µg/ml LPS and processed 1, 3, 6, and 24 h later for measurement of intracellular IL-1β, as detailed in the “Methods” section. Apo-SAA (■); LPS (▓). Data are expressed as mean ± sem, *n* = 3. The quantity of intracellular IL-1β in unstimulated cells was below the detection limit of the ELISA assay kit. For LPS, **p < 0.01 vs 3 and 6 h; for Apo-SAA, °°°*p* < 0.001 vs 1 h, ***p < 0.001 vs 3 and 24 h. (TIF 485 kb)

Additional file 3: **Figure S3.** Treatment of rat cortical microglia with LPS, in a time-dependent manner, upregulates mRNA for TLR2 and downregulates that for TLR4. Cultures were treated the day after plating with 0.1 μg/ml LPS and processed 1, 3, 6, and 24 h later for q-PCR, as detailed in the “Methods” section. (a) TLR2. (b) TLR4. Data are presented as relative expression level (normalized with respect to β-actin (βACT)) at each time point and are mean + sem, *n* = 3. Control (CTRL; untreated) cultures. Data are expressed as mean ± sem, *n* = 3 (*n* = 9 for 3 h). (a) **p < 0.01 vs CTRL (3 h) and ***p < 0.001 vs CTRL (6 h). (b) *p < 0.05 vs CTRL (3 h), ***p < 0.001 vs CTRL for that time point. Note the difference in expression levels between TLR2 and TLR4 mRNA. (TIF 957 kb)

## Abbreviations

AD: Alzheimer’s disease
DMEM: Dulbecco’s modified Eagle’s medium
FCS: Fetal calf serum
IL-1β: Interleukin-1β
L-LME: L-leucyl-L-leucine methyl ester
LPS: Lipopolysaccaride
MS: Multiple sclerosis
q-PCR: Quantitative real-time-PCR
TLR: Toll-like receptor
TNF-α: Tumor necrosis factor-α
