# Supplementary figures and images for: Serum amyloid A primes microglia for ATP-dependent interleukin-1β release
Source: J Neuroinflammation. 2018 May 26;15:164. doi: 10.1186/s12974-018-1205-6 (PMC5970445; doi:10.1186/s12974-018-1205-6)

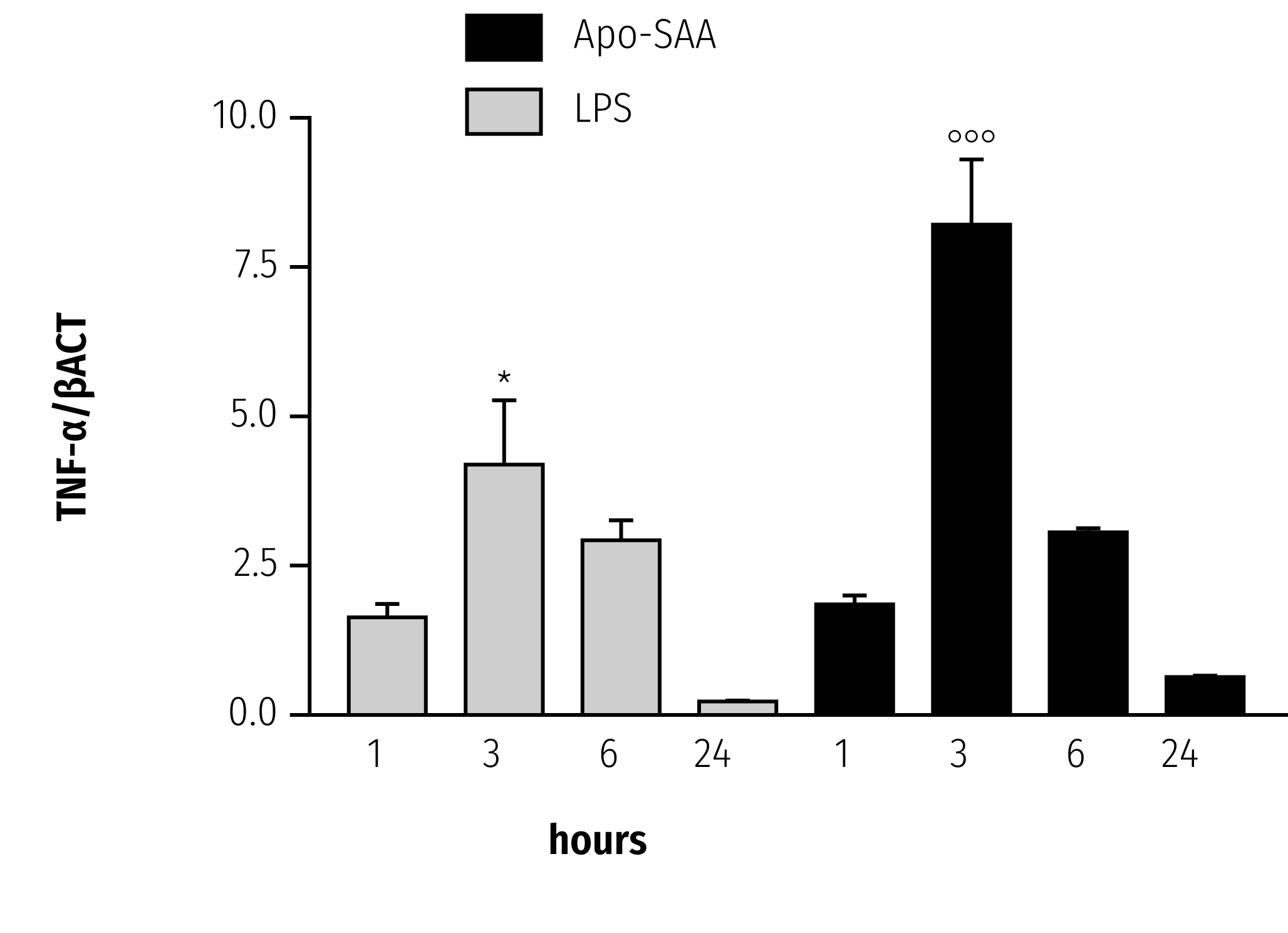

Supplement: Supplementary file 1 — Figure S1. Treatment of rat cortical microglia with Apo-SAA or LPS upregulates, in a time-dependent manner mRNA for TNF-α. Cultures were treated the day after plating with 0.5 μg/ml recombinant human Apo-SAA or 0.1 µg/ml LPS and processed 1, 3, 6, and 24 h later for q-PCR, as detailed in the “Methods” section. Data are presented as relative expression level (normalized with respect to β-actin (βACT)) at each time point and are mean ± sem, n = 3. Control values (which were at the limit of detection) were omitted for clarity. Apo-SAA (■); LPS (▓). For LPS, *p < 0.05 vs 1 and 24 h; for Apo-SAA: °°°p < 0.001 vs 1, 6, and 24 h. (TIF 468 kb) [file 12974_2018_1205_MOESM1_ESM.tif]

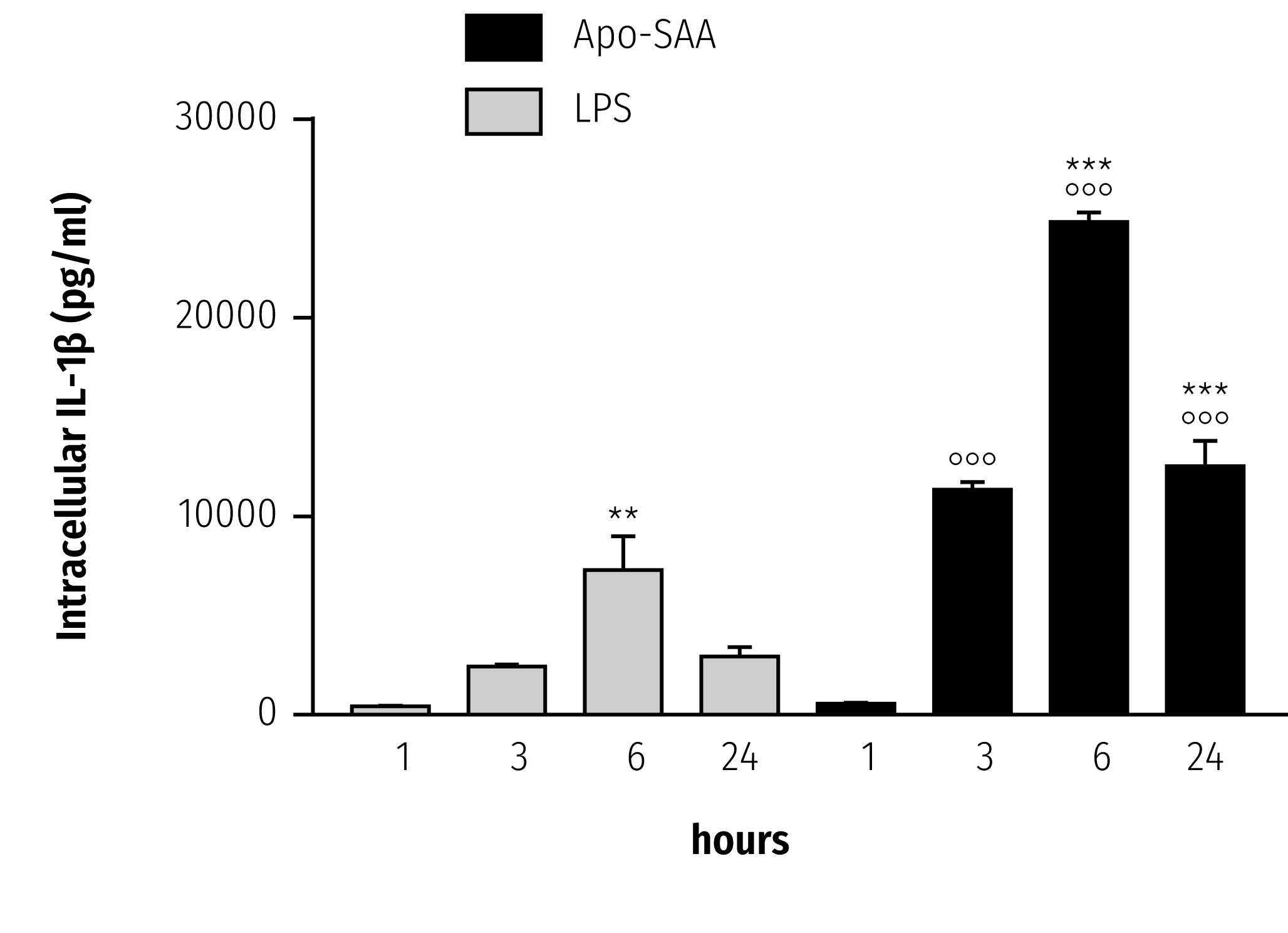

Supplement: Supplementary file 2 — Figure S2. Treatment of rat cortical microglia with Apo-SAA or LPS upregulates, in a time-dependent manner, intracellular content of IL-1β. Cultures were treated the day after plating with 0.5 μg/ml recombinant human Apo-SAA or 0.1 µg/ml LPS and processed 1, 3, 6, and 24 h later for measurement of intracellular IL-1β, as detailed in the “Methods” section. Apo-SAA (■); LPS (▓). Data are expressed as mean ± sem, n = 3. The quantity of intracellular IL-1β in unstimulated cells was below the detection limit of the ELISA assay kit. For LPS, **p < 0.01 vs 3 and 6 h; for Apo-SAA, °°°p < 0.001 vs 1 h, ***p < 0.001 vs 3 and 24 h. (TIF 485 kb) [file 12974_2018_1205_MOESM2_ESM.tif]

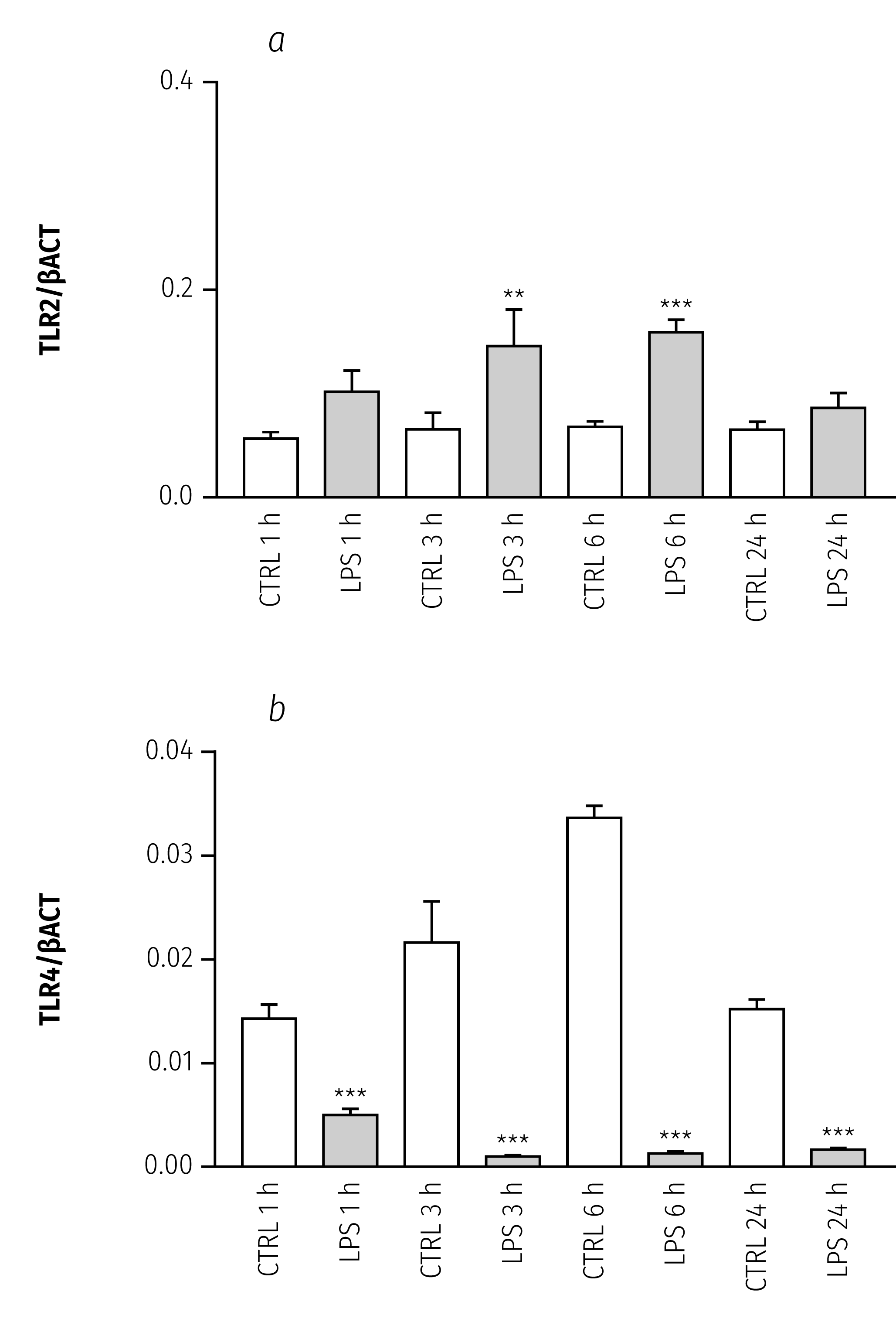

Supplement: Supplementary file 3 — Figure S3. Treatment of rat cortical microglia with LPS, in a time-dependent manner, upregulates mRNA for TLR2 and downregulates that for TLR4. Cultures were treated the day after plating with 0.1 μg/ml LPS and processed 1, 3, 6, and 24 h later for q-PCR, as detailed in the “Methods” section. (a) TLR2. (b) TLR4. Data are presented as relative expression level (normalized with respect to β-actin (βACT)) at each time point and are mean + sem, n = 3. Control (CTRL; untreated) cultures. Data are expressed as mean ± sem, n = 3 (n = 9 for 3 h). (a) **p < 0.01 vs CTRL (3 h) and ***p < 0.001 vs CTRL (6 h). (b) *p < 0.05 vs CTRL (3 h), ***p < 0.001 vs CTRL for that time point. Note the difference in expression levels between TLR2 and TLR4 mRNA. (TIF 957 kb) [file 12974_2018_1205_MOESM3_ESM.tif]
